# Corynoxeine Supplementation Ameliorates Colistin-Induced Kidney Oxidative Stress and Inflammation in Mice

**DOI:** 10.3390/antiox14050593

**Published:** 2025-05-15

**Authors:** Yue Liu, Ruichen Zhang, Tony Velkov, Jianzhong Shen, Shusheng Tang, Chongshan Dai

**Affiliations:** 1 National Key Laboratory of Veterinary Public Health and Safety, College of Veterinary Medicine, China Agricultural University, Beijing 100193, China; 2 Technology Innovation Center for Food Safety Surveillance and Detection (Hainan), Sanya Institute of China Agricultural University, Sanya 572025, China; 3 Department of Pharmacology, Biodiscovery Institute, Monash University, Parkville, VIC 3052, Australia

**Keywords:** colistin, nephrotoxicity, corynoxeine, oxidative stress, inflammatory response

## Abstract

This study investigated the protective effects of corynoxeine, a natural alkaline compound, on colistin-caused nephrotoxicity using a murine model. Forty mice were divided randomly into control, corynoxeine-only (20 mg/kg/day, intraperitoneal injection), colistin-only (20 mg/kg/day, intraperitoneal injection), and colistin (20 mg/kg/day) + corynoxeine (5 and 20 mg/kg/day) groups (8 mice in each group). All treatments were maintained for seven consecutive days. Results showed that colistin treatment at 20 mg/kg/day for seven days significantly increased serum urea nitrogen and creatinine levels and induced the loss and degeneration of renal tubular epithelial cells, which were markedly ameliorated by corynoxeine co-treatment at 5 or 20 mg/kg/day. Corynoxeine supplementation also markedly attenuated colistin-induced increases in malondialdehyde levels and decreases in reduced glutathione levels and superoxide dismutase and catalase activities in the kidneys. Furthermore, corynoxeine supplementation significantly decreased the expression of transforming growth factor β (TGF-β) and nicotinamide adenine dinucleotide phosphate hydrogen oxidase 4 (NOX4) proteins and nuclear factor kappa B (*NF-κB*), interleukin-1beta (*IL-1β*), *IL-6*, and tumor necrosis factor-α mRNAs, while it significantly increased the expression of erythroid 2-related factor 2 (Nrf2) and heme oxygenase-1 (HO-1) proteins in the kidneys. In conclusion, these results reveal that corynoxeine can protect against colistin-induced nephrotoxicity in mice by inhibiting oxidative stress and inflammation, which may partly be attributed to its ability on the activation of the Nrf2/HO-1 pathway and the inhibition of the TGF-β/NOX4 and NF-κB pathways.

## 1. Introduction

Polymyxins, such as polymyxin E (also called colistin) and polymyxin B, serve as critical last-resort treatments for the life-threatening infections caused by multidrug-resistant Gram-negative *Enterobacteriaceae* bacteria [1,2]. Despite their efficacy, the current dosing regimens of colistin often fail to achieve optimal plasma concentrations, necessitating higher doses for effective bacterial eradication and resistance prevention [3,4,5]. However, nephrotoxicity remains a significant barrier, affecting approximately 60% of patients and limiting its therapeutic use [5]. Consequently, the development of nephroprotective strategies to mitigate renal damage during polymyxin therapy is urgently required.

Recent advancements in immunohistochemical and microscopic analyses have demonstrated that polymyxins exhibit substantial accumulation in renal tissues [6,7]. This finding has prompted research into the molecular mechanisms responsible for kidney damage caused by polymyxins. Identifying the primary signaling pathways involved in the death of kidney tubular cells during polymyxin-induced nephrotoxicity is essential for creating agents that protect the kidneys [5]. In renal tubular cell cultures, exposure to polymyxin B and colistin results in a dose-dependent reduction in mitochondrial membrane potential, alterations in cellular morphology, and increased production of reactive oxygen species (ROS) [8]. Research indicates that the apoptosis triggered by colistin is significantly influenced by the transforming growth factor β (TGF-β)-regulated, nicotinamide adenine dinucleotide phosphate hydrogen oxidase 4 (NOX4)-dependent generation of ROS [8,9]. Numerous studies have explored the mechanisms behind colistin’s cytotoxic and nephrotoxic effects, highlighting the involvement of several pathways. These include the engagement of death receptor pathways, impaired mitochondrial function, endoplasmic reticulum stress, autophagy processes, mitogen-activated protein kinases (MAPKs), p53, mammalian target of rapamycin (mTOR)/protein kinase B (Akt), aryl hydrocarbon receptor (AhR)/cytochrome P450 family 1 subfamily A member 1 (CYP1A1), nuclear factor erythroid 2-related factor 2 (Nrf2)/heme oxygenase-1 (HO-1), signal transducer and activator of transcription 3 (STAT3), nuclear factor kappa B (NF-κB), and forkhead box protein O1 (FOXO1) pathways [10,11,12,13,14,15,16,17]. Understanding these diverse mechanisms provides insights into the complex cellular responses induced by colistin and suggests potential targets for therapeutic intervention to mitigate its adverse effects.

*Uncaria rhynchophylla* (*U. rhynchophylla*), commonly referred to as “Gou-teng” in traditional Chinese medicine, has a long history of use in treating various health conditions, including liver ailments, neurological disorders, and hypertension [18]. This herb, widely utilized in both China and Korea, has garnered significant attention in modern research for its diverse therapeutic properties. Numerous studies have highlighted its potent pharmacological effects, such as reducing inflammation, combating oxidative stress, and providing protection to the nervous system. These findings underscore its potential as a valuable natural remedy in contemporary healthcare practices [19]. For example, Xie et al. found that *U. rhynchophylla* extract supplementation via the oral administration at 0.78, 1.56, and 3.12 g/kg body weight per day for twenty-eight days can effectively inhibit the TGF-β, RhoA, and Rho-associated coiled-coil protein kinase 1 (ROCK1) proteins, following to inhibit angiotensin II-induced myocardial fibrosis in mice [20]. In another study, the major ingredient alkaloid compounds from *U. rhynchophylla* (3β-isodihydrocadambine, 22-O-Demethyl-22-O-β-glucopyranosyl isocorynoxeine, akuammigine pseudoindoxy, rhynchophylline N-oxide, rhynchophylline, isocorynoxeine, isorhynchophylline, corynoxeine, and hirsutine) supplementation at 60 and 120 mg/kg body weight for thirty days can effectively reduce the neuroinflammatory response and mitochondrial apoptotic pathway, then markedly reduce the brain injury in mice with Alzheimer’s disease [21]. Shin et al. showed that *U. rhynchophylla* extract supplementation at 100 and 200 mg/kg body weight markedly reduced thioacetamide exposure-caused acute liver toxicity via attenuating inflammatory response and oxidative stress [22]. Additionally, it was reported that the aqueous extract of *U. rhynchophylla* can effectively block the phosphorylation of MAPKs and Akt, block the activation of NF-κB, and finally reduce LPS-induced the release of inflammatory factors such as nitric oxide (NO) and interleukin-1beta (IL-1β) in macrophages [23].

**Figure 1:**
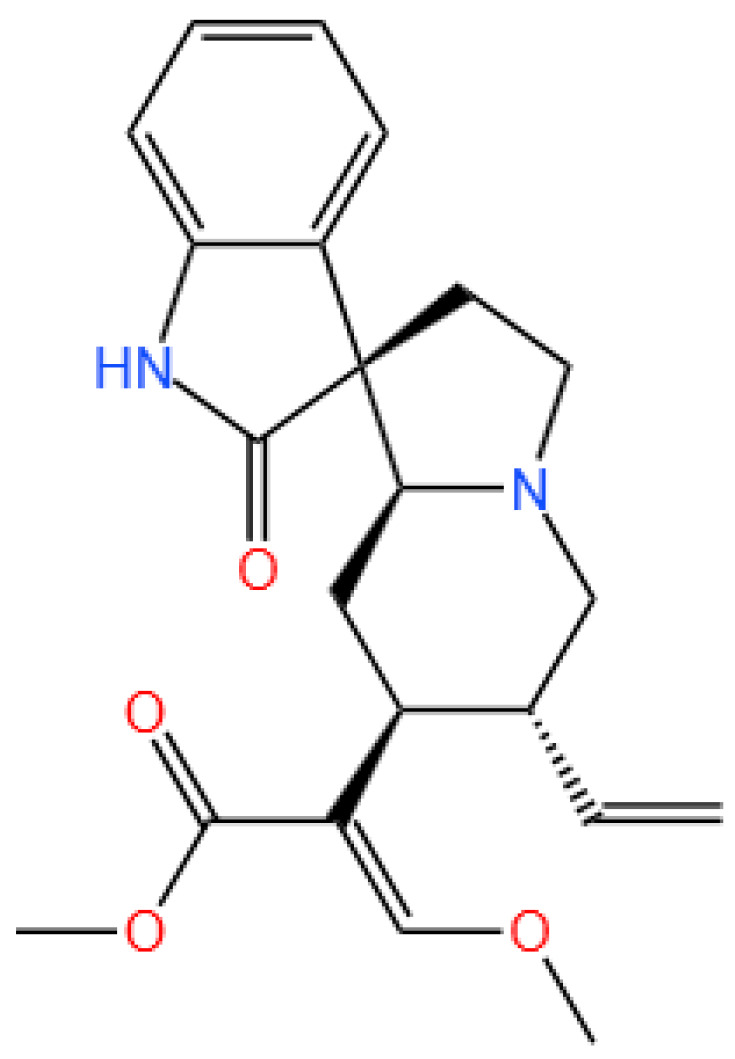
The structure of corynoxeine.

Corynoxeine (it was abbreviated Cory, and the structure was shown in Figure 1), an alkaloid with a tetracyclic indole structure, represents a significant bioactive component found in extracts derived from *U. rhynchophylla* [21,24]. Chen et al. found that oral supplementation of Cory at 2.5 and 5 mg/kg body weight per day for thirty-five days can markedly inhibit the production of tumor necrosis factor-α (TNF-α) and IL-8 proteins, reduce the activation of glial cells, and induce autophagy by downregulation of mTOR pathway, finally protecting against brain damage caused by rotenone in rats [25]. Recent studies have highlighted Cory’s capability as a suppressor within the MAPK/extracellular signal-regulated kinase 1/2 (ERK1/2) signaling cascade, effectively curtailing the proliferation of vascular smooth muscle cells in rat aortas and offering therapeutic prospects for hypertension management [26]. Notably, ERK1/2 is implicated in mediating inflammatory responses and renal damage induced by colistin [27,28]. Additionally, rhynchophylline, another significant component derived from *U. rhynchophylla*, has demonstrated robust antioxidant and anti-inflammatory properties through the stimulation of the Nrf2/ARE pathway and suppression of the NF-κB pathway [29,30]. Despite these advancements, the specific role of Cory in protecting against colistin-associated nephrotoxicity remains unexplored. This investigation seeks to elucidate the nephroprotective efficacy of Cory against colistin-induced kidney damage, focusing on the modulation of oxidative stress indicators, inflammatory markers, and the involvement of the TGF-β/NOX4, Nrf2, and NF-κB pathways.

## 2. Materials and Methods

### 2.1. Chemicals and Reagents

Cory (CAS 630-94-4) was sourced from Yuanye Bio-Technology (Shanghai, China). Colistin sulfate (CAS 1264-72-8; purity ≥ 19,000 IU/mg), phenylmethylsulfonyl fluoride (PMSF), and dimethyl sulfoxide (DMSO) were acquired from Sigma-Aldrich’s branch in Shanghai (China). Sodium fluoride (NaF) and sodium orthovanadate (Na_3_VO_4_) were procured from Beyotime (Beijing, China). All remaining chemicals utilized in the study met or exceeded analytical grade standards.

### 2.2. Animals and Experiment Designs

All research involving animal subjects was authorized by the Institutional Animal Ethics Review Board at China Agricultural University (Research license AW40114202-2-02, approval date 4 November 2024). This study strictly adhered to the established standards for humane animal research practices as outlined in China Agricultural University’s animal welfare policy documents.

Male C57BL/6 mice, aged 8 weeks with body weights ranging between 20 and 23 g, were sourced from Beijing Vital River Laboratory Animal Technology Co., Ltd. (Beijing, China). The housing conditions were meticulously controlled to ensure optimal environmental parameters: temperature was maintained between 22 and 24 °C, humidity levels were kept at 50–60%, and a consistent 12 h light/dark cycle was implemented. The mice were provided with unrestricted access to a standard pellet diet (Beijing Vital River Laboratory Animal Technology Co., Ltd., Beijing, China) and clean drinking water to support their nutritional needs. After 1 week adaptation, forty mice were randomly assigned into control group, corynoxeine-only (i.e., Cory 20 mg/kg/day) group, colistin-only (i.e., colistin 20 mg/kg/day) group, colistin (20 mg/kg/day) + low-dose corynoxeine (5 mg/kg/day) (i.e., colistin + Cory 5 mg/kg/day) group, and colistin (20 mg/kg/day) + high-dose corynoxeine (20 mg/kg/day) (i.e., colistin + Cory 20 mg/kg/day) group, with eight mice in each group. In the control group, mice were orally administered an equal volume of 1% DMSO and two i.p. injections of saline. In all the colistin groups, mice were injected intraperitoneally (i.p.) with colistin (sulfate) at 20 mg/kg bodyweight per day (it was divided into 2 doses, at least 8 h apart), according to our previous studies with minor revision [1]. Cory was dissolved in 1% DMSO and i.p. 2 h prior to colistin administration. The dose selection of Cory mainly referred to the previous studies about *U. rhynchophylla* total alkaloid and in animal experiments [31,32]. All mice were treated for seven days. At 12 h after the last dose, mice were anesthetized and euthanized using i.p. a higher dose of sodium pentobarbital at 80 mg/kg body weight. Then, the serum samples were isolated for biochemical analysis. The kidneys are first soaked in liquid nitrogen and then moved to a −80 freezer for storage for protein and gene expression analysis or immersed in a 4% paraformaldehyde solution for histopathology analysis.

### 2.3. Serum Isolation and Biochemical Analysis

To obtain the serum fraction for subsequent analysis, the collected blood specimens from the experimental rodents were processed through a centrifugation protocol at 3000× *g* for a duration of 20 min under ambient laboratory conditions. The levels of serum creatinine (CRE) and urea nitrogen (UN) were measured using commercial CRE and UN reagent kits (Beijing Lidman Biochemical Co., Ltd., Beijing, China) by using a Hitachi 7080 Automated Biochemical Analyzer manufactured by Hitachi Ltd. (Tokyo, Japan).

### 2.4. Oxidative Stress Biomarker Measurement

In this study, segments of renal tissue were carefully extracted and processed to create a 10% (*w*/*v*) homogenate, adhering to methodologies established in prior research [33]. The quantification of protein levels within the supernatant was determined using a bicin-choninic acid (BCA) Protein Assay Kit (Thermo Fisher Scientific, Waltham, MA, USA). To assess oxidative stress parameters, measurements of the enzymatic activities of catalase (CAT), superoxide dismutase (SOD), malondialdehyde (MDA), and reduced glutathione (GSH) levels were conducted using specialized assay kits provided by the Nanjing Jiancheng Institute of Biological Engineering (Nanjing, China). All procedures were meticulously executed in compliance with the manufacturers’ guidelines (shown in Supplementary Materials and Methods).

### 2.5. Measurement of the Activities of Caspases-9 and -3

The enzymatic activities of caspase-9 and caspase-3 in mice kidney tissues were quantified through the utilization of commercially available assay kits (Beyotime, Beijing, China). The experimental procedures were meticulously executed in strict compliance with the manufacturer’s established guidelines (shown in Supplementary Materials and Methods). The quantification of protein levels was performed utilizing a BCA™ protein determination kit (Beyotime, Beijing, China). All experimental results pertaining to caspase-9 and caspase-3 enzymatic activities were subsequently normalized based on the quantified protein concentrations detected in corresponding samples.

### 2.6. Histopathological Examination

Five kidney samples were randomly selected from each group. Renal tissue samples were carefully excised and immersed in a 4% paraformaldehyde solution, maintaining a volume approximately tenfold that of the tissue specimens. After a 48 h fixation process, the samples underwent sequential dehydration using an ascending series of ethanol concentrations (ranging from 70% to 100%). The dehydrated tissues were then embedded in paraffin and sliced into 5 μm sections. These sections were subsequently subjected to hematoxylin–eosin (H&E) staining for microscopic evaluation (Leica Microsystems, Wetzlar, Germany). A semi-quantitative scoring system (SQS), previously established in published literature [1], was employed to quantify these morphological changes. The specific protocol and implementation details of the SQS methodology are comprehensively documented in the Supplementary Materials and Methods section.

### 2.7. Immunohistochemical Examination

After conducting the histopathological evaluation as described earlier, the paraffin-embedded kidney sections were subjected to streptavidin—biotin—peroxidase staining for immunohistochemical assays, according to our published protocols [33]. Rabbit polyclonal antibodies targeting NOX4 and TGF-β (both diluted at 1:200; ProteinTech Group, Inc., Chicago, IL, USA) were utilized. The detail protocols were shown in the Supplementary Materials and Methods.

### 2.8. Quantitative Real-Time PCR Analysis

Total RNA was isolated from kidney tissue samples utilizing the Total RNA Extraction Kit (Tiangen Biotech, Beijing, China; Product No. 4992858), following the manufacturer’s protocol meticulously. The purity of the extracted RNA was evaluated by determining the absorbance ratio at 260/280 nm, with all samples exhibiting ratios between 1.9 and 2.1. Approximately 1 μg of total RNA was reverse transcribed into complementary DNA (cDNA) using the HiScript^®^ III All-in-One RT SuperMix (Vazyme Biotech Co., Ltd., Nanjing, China). Subsequently, quantitative real-time PCR was performed to quantify the mRNA levels of *NF-κB*, *IL-1β*, *IL-6*, and *TNF-α*, employing the SYBR Green Master Mix (Vazyme Biotech Co., Ltd.). The specific primer sequences used in this study are detailed in Supplementary Table S1. Standard cycling conditions were used, including a preamplification step of 95 °C for 5 min, followed by amplification for 40 cycles of 95 °C for 20 s, 60 °C for 1 min, and 72 °C for 30 s, and β-actin was selected as the internal reference gene for normalization. Relative gene expression levels were calculated using the comparative ΔΔCt method. Each sample is subjected to three repeated techniques.

### 2.9. Western Blot Analysis of Kidney Tissue Proteins

Four kidney samples were randomly selected from each group. Each sample was prepared through a series of steps including mechanical scraping, ultrasonic disruption, centrifugal separation, and dissolution in RIPA lysis buffer (Beyotime, Beijing, China), according to our previous description [33]. The extraction buffer was enhanced with a cocktail of protease and phosphatase inhibitors (comprising PMSF at 10 μg/mL, Na_3_VO_4_ at 0.5 mM, and NaF at 50 mM). Quantitative assessment of protein content was performed using a BCA™ protein determination kit. Regarding the primary antibodies, rabbit antibodies directed against Nrf2 (Cat No.12721; diluted at 1:1000; Cell Signaling Technology, Danvers, MA, USA), HO-1 (Cat No. 10701-1-AP), B-cell lymphoma 2 (Bcl-2) (Cat No. 26593-1-AP), Bcl-2 associated X (Bax; Cat No. 50599-2-Ig) (all antibodies diluted at 1:1000; ProteinTech Group, Inc., Chicago, IL, USA), and mouse monoclonal antibodies against β-actin (Cat No. sc-58673; diluted at 1:1000; Santa Cruz Biotechnology, Dallas, TX, USA) were utilized. Horseradish peroxidase-conjugated goat anti-rabbit (Cat No. A0208) or anti-mouse (Cat No. A0216) IgG (both diluted at 1:5000; Beyotime, Beijing, China) were employed as the secondary antibodies. The detailed procedures for Western Blotting adhered to those of our previous study [34]. A Tanon Chemiluminescent Imaging System (Shanghai, China) was used to capture images of the protein blot. Eventually, the expression levels of all proteins were analyzed with ImageJ software (V1.8.0.112, NIH, Bethesda, MD, USA).

### 2.10. Statistical Analysis

The statistical evaluation and graphical representation of the dataset were conducted through the utilization of GraphPad Prism version 10.0 (Graph Pad Software, Inc., San Diego, CA, USA). Quantitative findings are expressed as arithmetic averages accompanied by standard deviation (S.D.) values, except in instances where alternative representations are specified. For homogeneity assessment across multiple groups, one-way ANOVA was implemented, while inter-group variations were examined using Tukey’s post hoc analysis. A value of *p* < 0.05 was deemed to possess statistical significance.

## 3. Results

### 3.1. Cory Supplementation Ameliorates Colistin-Induced Nephrotoxicity in Mice

**Figure 2:**
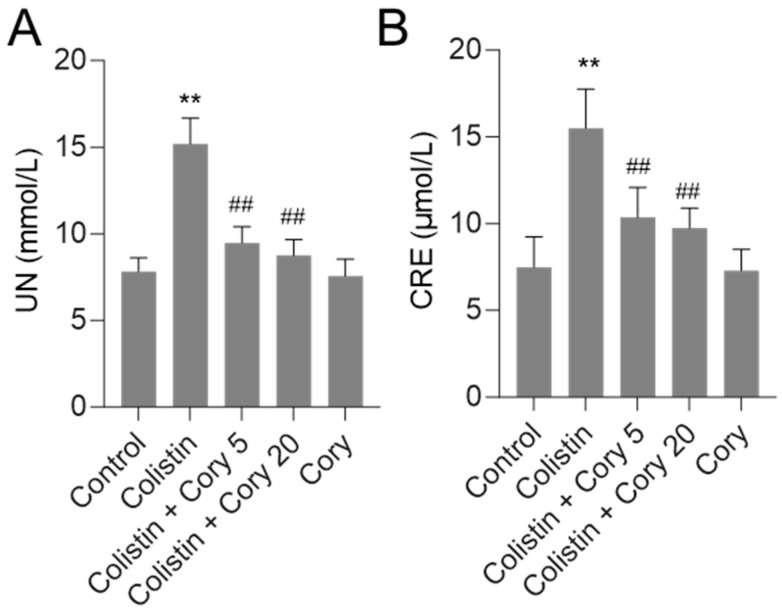
Alterations in serum urea nitrogen (UN (**A**)) and creatinine (CRE (**B**)) concentrations. Data were presented as mean ± S.D. (n = 8). ** *p*  <  0.01, compared to control. ^##^ *p*  <  0.01, compared to colistin-only group.

As shown in Figure 2, in the colistin-only treatment group, the levels of serum UN and CRE were significantly increased to 15.2 mmol/L and 15.5 μmol/L (both *p* < 0.01), respectively, compared to those in the control group. Cory pre-treatment at 5 or 20 mg/kg/day significantly decreased serum UN and CRE levels. In the colistin + Cory 5 mg/kg/day group, the serum UN and CRE levels decreased to 9.5 mmol/L and 10.4 μmol/L, respectively; in the colistin + Cory 20 mg/kg/day group, the serum UN and CRE levels decreased to 8.7 mmol/L and 9.7 μmol/L, respectively, compared to those in the colistin model group. In the Cory 20 mg/kg/day group, the levels of serum UN and CRE have no marked changes compared to the control group.

**Figure 3:**
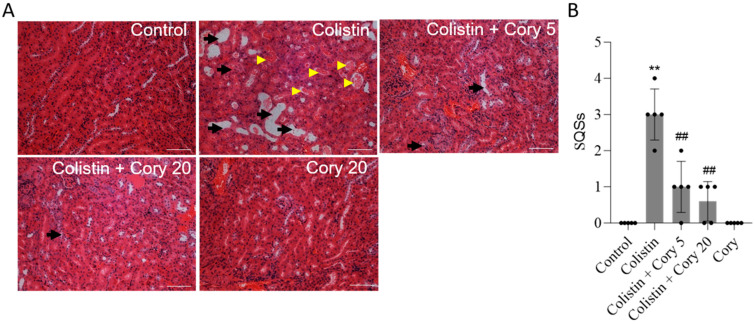
Representative histopathological changes and semi-quantitative analysis. The kidney slices were stained with the hematoxylin and eosin (H&E) method. (**A**) The representative images of histopathological changes in kidneys of mice. (**B**) SQS values for each group. The results were shown as mean ± S.D. (n  =  5). ** *p * <  0.01, compared to the untreated control. ^##^ *p*  <  0.01, compared to colistin-only group. Bar = 50 μm. Yellow arrowheads indicate cast formation; black arrows indicate marked tubular degeneration, necrosis, and tubular dilation.

The histopathological changes in the kidney tissue were further performed. As shown in Figure 3, colistin treatment induced marked tissue damage, which was evident by the marked tubular degeneration, cast formation, tubular dilation, and necrosis (Figure 3A); correspondingly, the SQS scores increased to 3 (*p* < 0.01). Cory supplementation can markedly attenuate colistin treatment-induced kidney tissue damage. In the colistin + Cory 5 and 20 mg/kg/day group, the SQS scores were decreased to 1 and 0.6 (both *p* < 0.01), respectively (Figure 3B). Compared to the control group, the kidney in the Cory-only group had no marked histopathological changes (Figure 3).

### 3.2. Cory Supplementation Ameliorates Colistin Treatment-Caused Oxidative Stress Damage in the Kidneys of Mice

**Figure 4:**
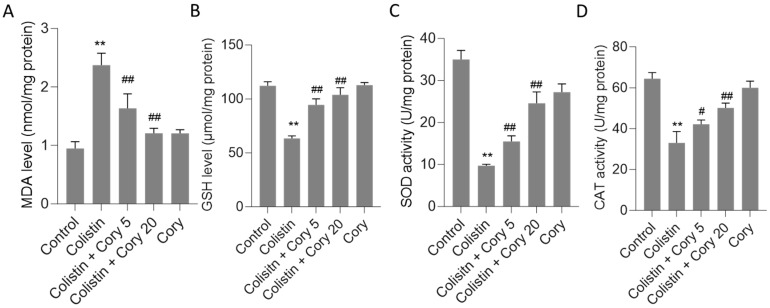
The changes of oxidative stress markers in the kidney tissues of mice: (**A**) MDA levels; (**B**) GSH levels; (**C**) SOD activities; and (**D**) CAT activities. Data were mean ± S.D. (n  =  8). ** *p*  <  0.01, compared to the untreated control. ^#^ *p*  <  0.05 and ^##^ *p * <  0.01, compared to the colistin-only treatment group.

This study evaluated several oxidative stress biomarkers, including GSH and MDA, as well as SOD and CAT activities. As depicted in Figure 4, the administration of colistin led to significant alterations in these markers compared to the untreated control group. Specifically, MDA levels rose sharply to 2.4 nmol/mg protein (*p* < 0.01) (Figure 4A), while GSH levels dropped significantly to 63.3 μmol/mg protein (*p* < 0.01) (Figure 4B). Concurrently, CAT and SOD activities were markedly reduced to 33.1 U/mg protein (*p* < 0.01) and 9.7 U/mg protein (*p* < 0.01), respectively (Figure 4C,D). However, Cory supplementation offered a substantial ameliorative effect on the oxidative stress induced by colistin. In the group treated with colistin plus Cory 20 mg/kg/day, MDA levels decreased to 1.2 nmol/mg protein, GSH levels increased to 103.9 μmol/mg protein, and CAT and SOD activities rose to 50.1 U/mg protein and 24.6 U/mg protein, respectively. Importantly, when compared to the untreated control group, the levels of MDA and GSH, along with the activities of SOD and CAT, remained largely unchanged (Figure 4).

### 3.3. Cory Supplementation Attenuates Colistin Treatment-Caused Activation of the Mitochondrial Apoptotic Pathway

**Figure 5:**
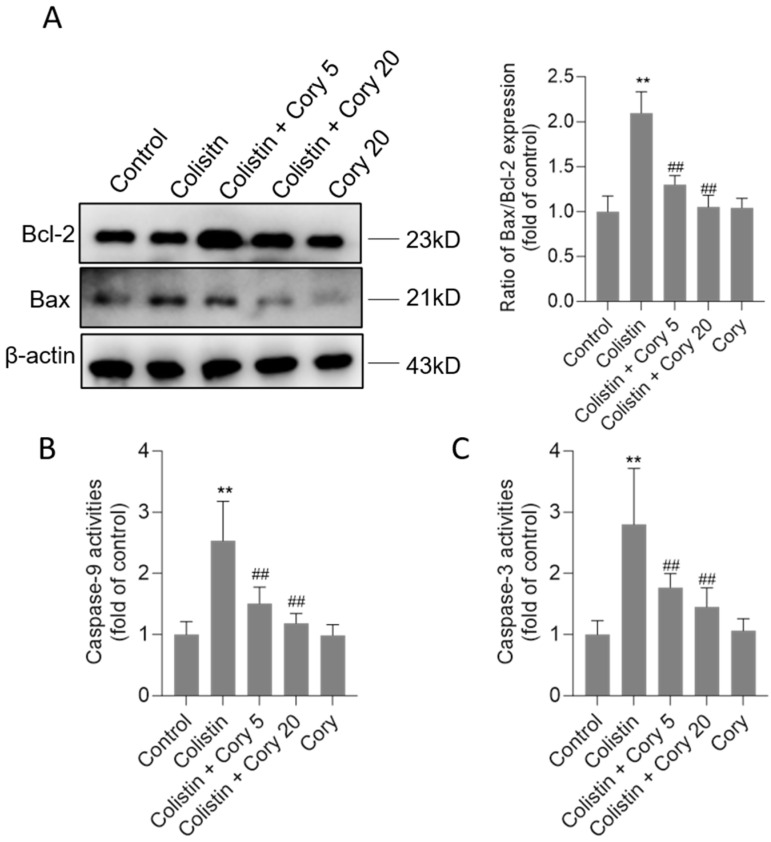
The changes of Bax/Bcl-2 ratio and activities of caspases-9 and -3 in the kidney tissues of mice. (**A**) Representative image of Western Blot (left) and its quantitative analysis (right). (**B**) The activities of caspase-9. (**C**) The activities of caspase-3. Data were shown as mean ± S.D. (n  =  8). ** *p*  <  0.01, compared to the untreated control. ^##^ *p * <  0.01, compared to the colistin-only treatment group.

Compared to the untreated control group, the exposure to colistin significantly upregulated Bax protein expression and downregulated Bcl-2 protein expression. As shown in Figure 5, colistin treatment increased the ratio of Bax/Bcl-2 expression to 2.1-fold (*p* < 0.01). Cory supplementation markedly revised these changes. In the colistin + Cory 5 and colistin + Cory 20 groups, the ratio of Bax/Bcl-2 expression decreased to 1.3- and 1.1-fold, respectively. Furthermore, Cory supplementation markedly reduced colistin treatment-induced caspase activation. In the colistin + Cory 5 and colistin + Cory 20 groups, the levels of caspase-9 decreased from 2.5-fold to 1.5- and 1.2-fold, and the levels of caspase-3 decreased from 2.8-fold to 1.8- and 1.4-fold, respectively (Figure 5B,C). In the Cory-only treatment group, there was no marked changes in the ratio of Bax/Bcl-2 expression and the activities of caspases-9 and -3 (Figure 5).

### 3.4. Cory Supplementation Downregulated the Expression of TGF-β and NOX4 Proteins in the Kidneys

**Figure 6:**
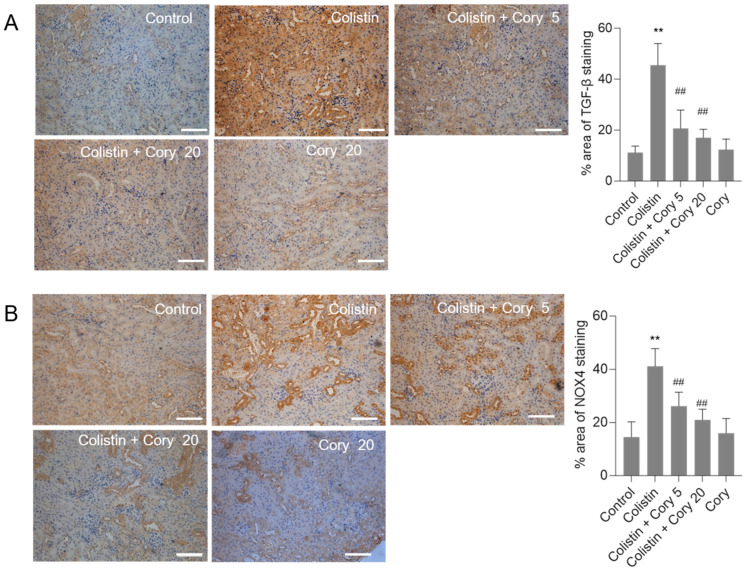
The expression changes of TGF-β and NOX4 proteins in the kidneys of mice. (**A**) Representative image of immunohistochemical staining. (**B**) The quantitative analysis of immunohistochemical staining. Data were shown as mean ± S.D. (n  =  5). ** *p*  <  0.01, compared to the untreated control. ^##^ *p * <  0.01, compared to the colistin-only treatment group. Bar = 50 μm.

The expression of TGF-β and NOX4 proteins in the kidneys of mice were quantitatively analyzed using the immunohistochemical staining. Compared to the untreated control group, colistin treatment significantly increased the levels of TGF-β (Figure 6A) and NOX4 (Figure 6B) proteins. Cory supplementation markedly decreased the expression of TGF-β and NOX4 proteins. In the colistin + Cory 20 group, the TGF-β and NOX4 proteins decreased from 45.5% and 41.2% to 17.3% and 21.4% (both *p* < 0.01), respectively. There were no marked changes in the expression of TGF-β and NOX4 proteins, compared to those in the untreated control group (Figure 6).

### 3.5. Cory Supplementation Upregulated the Expression of Nrf2 and HO-1 Proteins in the Kidneys

**Figure 7:**
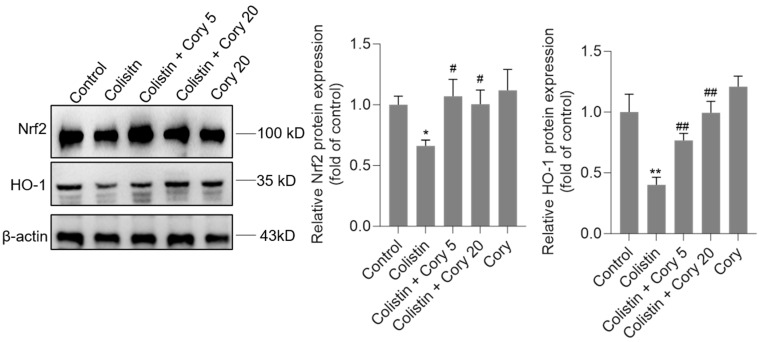
The expression of Nrf2 and HO-1 proteins in the kidneys of mice. Representative image of Western Blot (**left**) and the quantitative analysis (**right**). Data were shown as mean ± S.D. (n  =  4). * *p*  <  0.05 and ** *p*  <  0.01, compared to the untreated control. ^#^ *p*  <  0.05 and ^##^ *p * <  0.01, compared to the colistin-only treatment group.

As illustrated in Figure 7, the administration of colistin resulted in a notable reduction in the levels of Nrf2 and HO-1 proteins in the renal tissues of experimental mice, decreasing to 0.67- and 0.41-fold (*p*  <  0.05 or 0.01), respectively, when compared to the control group. However, Cory supplementation led to a significant upregulation of these proteins. Specifically, within the colistin + Cory 20 mg/kg group, Nrf2 and HO-1 protein expressions increased to 1.0-fold (*p*  < 0.01) and 0.98-fold (*p*  < 0.01), respectively. Additionally, a marginal enhancement in the expression of Nrf2 and HO-1 proteins was observed in the group that received only Cory at 20 mg/kg body weight, relative to the untreated control group (Figure 7).

### 3.6. Cory Treatment Attenuates the Expression of NF-κB, IL-1β, IL-6, and TNF-α mRNAs in the Kidney Tissues

**Figure 8:**
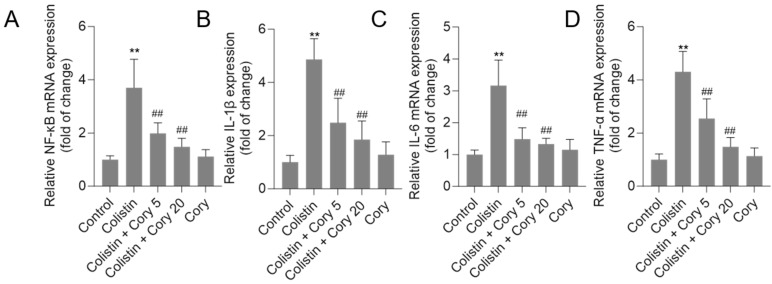
The expression of *NF-κB* (**A**), *IL-1β* (**B**), *IL-6* (**C**), and *TNF-α* (**D**) mRNAs in the kidney tissues of mice to colistin or colistin + Cory treatments. Data were shown as mean ± S.D. (n  =  8). ** *p*  <  0.01, compared to the untreated control. ^##^ *p * <  0.01, compared to the colistin-only treatment group.

As illustrated in Figure 8, treatment with colistin resulted in significant increases in *NF-κB*, *IL-1β*, *IL-6*, and *TNF-α* mRNA levels, reaching 3.7-, 4.8-, 3.2-, and 4.3-fold (all *p* < 0.01) increases, respectively. Notably, co-administration of Cory supplementation effectively countered these colistin-induced effects. In particular, the colistin + Cory 20 mg/kg/day group showed marked reductions in the expression of these inflammatory markers, with *NF-κB*, *IL-1β*, *IL-6*, and *TNF-α* mRNA levels decreasing to 1.5-, 1.9-, 1.3-, and 1.5-fold (all *p* < 0.01), respectively. Importantly, when administered alone at a dosage of 20 mg/kg/day for 7 days, Cory did not exhibit any significant impact on the baseline expression of these inflammatory markers compared to the control group.

## 4. Discussion

The emergence of multidrug-resistant Gram-negative bacteria has escalated into a global healthcare crisis, particularly within hospital settings. These pathogens spread swiftly and leave clinicians with limited therapeutic options. Among the few antibiotics still effective against these “superbugs” are Polymyxin B and colistin. However, their clinical utility is hindered by significant dose-limiting nephrotoxicity, a critical barrier to successful treatment. Developing strategies to reduce this adverse effect and enhance the safety of polymyxin-based therapies is a pressing priority.

Cory stands out as a primary bioactive compound found within the extract of *U. rhynchophylla*. Research has demonstrated that Cory, along with the overall *U. rhynchophylla* extract, exhibits significant therapeutic properties [26,35,36,37,38]. These include robust anti-inflammatory effects, the ability to combat oxidative stress, and the prevention of apoptosis. These beneficial actions are achieved through the modulation of several critical signaling pathways, including NF-κB, Nrf2/HO-1, and MAPK/ERK. Such diverse mechanisms make Cory a promising candidate for further exploration in the realm of natural therapeutics [22,39,40]. Current results found that Cory supplementation at 5 and 20 mg/kg per day for seven days can both attenuate colistin-induced increases in the levels of serum CRE and UN and renal histopathological damage using a mouse model (Figure 2 and Figure 3), indicating the potential nephroprotective effects. Furthermore, the underlying molecular mechanisms were further investigated. Results from Figure 4, Figure 5, Figure 6, Figure 7 and Figure 8 showed that the nephroprotective effects of Cory involve the suppression of oxidative stress and NF-κB and TGF-β/NOX4 pathways and the activation of Nrf2/HO-1 pathways.

Recent studies have highlighted the significant contribution of oxidative stress in colistin-caused nephrotoxicity, both in proximal tubular cells’ cultures and rodent models [1,17,33,41,42,43]. Edrees et al. found that colistin treatment at 300,000 IU colistin/kg per day (equal to 10 mg/kg per day) for 6 days can significantly increase the levels of MDA, a marker of lipid peroxidation, and decrease GSH levels and CAT and SOD activities in the kidneys of rats [17]. In line with these findings, our research demonstrated increased MDA levels, reduced activities of CAT and SOD, and lower GSH levels in colistin-treated kidneys of mice (Figure 4). Cory supplementation at 5 or 20 mg/kg per day for seven days can markedly reverse the decreases in SOD and CAT activities and GSH levels as well as the increases in MDA levels caused by colistin exposure (Figure 4). This indicates that Cory supplementation can suppress colistin-induced oxidative damage in the kidneys via inhibiting lipid peroxidation and upregulating antioxidant enzymes activities and antioxidant levels. Notably, Cory supplementation at 20 mg/kg per day slightly increased MDA levels and decreased SOD and CAT activities, indicating that high doses of Cory supplementation may cause potential adverse effects (Figure 4). Therefore, more investigations on dose optimization are still needed.

Colistin treatment can induce cell apoptosis in various tissues (including kidney and brain, lung, and testes tissues) and cell lines (including PC12, N2a, HK-2, and A549 cells) [9,10,41,44,45,46,47,48,49]. Previous studies reported that colistin treatment at 15 mg/kg body weight per day for seven days can activate death receptor (up-regulation of Fas-associated death domain, Fas, and FasL), mitochondrial (up-regulation of cytochrome C and the ratio of Bax/Bcl-2), and endoplasmic reticulum (up-regulation of caspase-12, CHOP, ATF4, ATF6, and Grp78/Bip) pathways to induce cell apoptosis in animals’ kidneys and cultured renal tubular cells [33,50,51,52]. It is known that caspase-3 usually serves as a crucial mediator of apoptosis [53]. It can be activated through two distinct routes: the intrinsic (mitochondrial) pathway and the extrinsic (death receptor) pathway. Meanwhile, caspase-9 acts as a significant mediator within the mitochondrial apoptosis pathway [1,54]. The pan-caspase inhibitor Z-VAD-FMK can markedly attenuate colistin-induced cell apoptosis [46]. Several small compounds such as baicalein, lycopene, minocycline, and curcumin supplementation can effectively reduce the activities of caspases-9 and -3, following to ameliorate colistin-induced cell apoptosis [1,16,17,47,55]. In the present study, our data also showed that colistin treatment can significantly upregulate the ratio of Bax/Bcl-2 in the kidney tissues (Figure 5A). In addition, we also found that the marked increased caspases-9 and -3 activities in colistin-treated kidney tissues were detected (Figure 5B,C). Cory co-treatment can significantly decrease the ratio of Bax/Bcl-2 and caspases-9 and-3 activities (Figure 5B,C). Taken together, these data indicated Cory supplementation can inhibit colistin-induced apoptosis in kidney tissues of mice via the inhibition of mitochondrial apoptotic pathway.

Several previous studies have demonstrated that the activation of TGF-β/NOX4 pathways plays a critical role in colistin-induced oxidative stress [8,9,56]. Previous studies have identified that NOX4 is one of the major sources of endogenous ROS production and partly mediated TGF-β-mediated inflammatory and fibrotic responses [57]. In cultured human A549 and HK-2 cells, colistin treatment can dose-dependently increase the expression of TGF-β mRNAs and NOX4 proteins [8,9]. Inhibition of TGF-β or NOX4 using the specific inhibitors can effectively attenuate colistin treatment-induced production of ROS and cell apoptosis [8,15]. This evidence indicated that TGF-β and NOX4 proteins are two critical targets in the process of colistin-induced cytotoxicity and nephrotoxicity. In the present study, we also found that colistin treatment significantly increased the levels of TGF-β or NOX4 proteins in the kidney tissues of mice, and these increased changes were markedly attenuated in Cory supplementation groups (Figure 6). A previous study also reported that *U. rhynchophylla* extract supplementation also significantly inhibited the expression of TGF-β in the heart tissues, then partly attenuated an angiotensin II-induced myocardial fibrosis in mice [20]. Taken together, these results indicated that Cory co-treatment can inhibit colistin-induced oxidative stress damage in the kidneys, which may partly be dependent on its inhibitory activities on the TGF-β/NOX4 pathway. Moreover, the current study also indicated that Cory may be a key active substance that mediated *U. rhynchophylla* extract on the inhibitory effects of TGF-β.

Nrf2, a transcription factor, plays a central role in regulating genes related to the oxidative stress response [58,59]. When Nrf2 gets activated, it can effectively alleviate cellular oxidative damage [58,59]. This occurs through the activation of genes encoding Phase II detoxifying enzymes and antioxidant enzymes like glutathione peroxidase (GPX), HO—1, SOD, and CAT. The activation of the Nrf2/HO-1 pathway is a well-documented mechanism that contributes significantly to the protective effects against various forms of nephrotoxicity, including cadmium- and aflatoxin B1-induced nephrotoxicity [60,61]. Wang et al. reported that colistin treatment at 15 mg/kg body weight per day for seven days markedly downregulated the expression of Nrf2 and HO-1 protein expression in the kidney tissues, then induced renal oxidative stress and apoptosis [62]. Several studies reported that pharmacological activation of the Nrf2/HO-1 pathway by 7-hydroxycoumarin, caffeic acid, lycopene, and baicalein supplementation significantly attenuated colistin-induced renal oxidative stress and apoptosis via the activation of the Nrf2/HO-1 pathway in vitro and in vivo [1,14,16,62]. In the present study, our results showed that Cory supplementation can effectively recover the decreases in Nrf2 and HO-1 proteins caused by colistin in the kidney tissues of mice (Figure 7). Zhang et al. reported that alkaloids extracted from *U. rhynchophylla* at 20–60 mg/kg body weight per day for 3 weeks can effectively activate the Nrf2/HO-1 pathway, then partly contribute the protective effects on 1-Methyl-4-phenyl-1,2,3,6-tetrahydropyridine hydrochloride exposure-induced brain oxidative injury in mice [39]. Correctly, these findings indicated that the activation of the Nrf2/HO-1 pathway by Cory partly contributed to explain its protective effects on colistin-induced oxidative damage in the kidneys.

Inflammatory response is an important pathological feature of colistin- or polymyxin B-induced kidney injury [17,28,63]. Several studies have demonstrated that the colistin-induced pro-inflammatory response involves MAPK/ERK, NLRP3, and NF-κB pathways [1,17,28,55]. Of note, NF-κB is a mast transcriptional mediator of the pro-inflammatory cytokine response, which can transcriptionally regulate over 200 inflammation-related factors, such as *TNF-α*, *IL-1β*, *IL-6*, *IL-12*, and cyclooxygenase-2 (*COX2*) [64]. Suk et al. found that rats that were administered intraperitoneally with *U. rhynchophylla* extracts at 100–1000 mg/kg can dose-dependently protect against global cerebral ischemia-induced increases in COX2 and prostaglandin E_2_ in hippocampal neurons [65]. Similarly, *U. rhynchophylla* extract treatments can also markedly inhibit the production of nitric oxide (NO) and TNF-α in BV-2 mouse microglial cells in vitro [65]. In line with these published studies, current results showed that colistin treatment at 20 mg/kg/day for 7 day markedly increased the mRNA levels of *NF-κB* genes and its downstream genes’ mRNA expression, including *TNF-α*, *IL-1β*, and *IL-6* mRNAs (Figure 8). Cory supplementation can effectively attenuate colistin-induced expression of *NF-κB*, *TNF-α*, *IL-1β*, and *IL-6* mRNAs (Figure 8). These data suggested that Cory supplementation can attenuate colistin-induced nephrotoxicity via the inhibition of NF-κB mediated inflammatory response. It is also indicated that Cory may be one of key active substances during *U. rhynchophylla* extract-mediated anti-inflammatory activities. Furthermore, the activation of NF-κB may be modulated by upstream signaling pathways, such as ROS, TGF-β, MAPK/ERK, and Nrf2 pathways [64,66,67,68]. For example, Nrf2-mediated transcriptional expression of HO-1 can markedly inhibit the nuclear translocation of NF-κB and exhibited potent anti-inflammatory activities [69,70]. Therefore, the inhibition of Cory on the TGF-β/NOX4 pathway and the activation of Nrf2/HO-1 may also contribute to its inhibitory effects on the NF-κB-mediated inflammatory response caused by colistin treatment in the kidneys of mice. The precise molecular mechanisms still need more investigation.

In the present study, we only tested the protective effects of Cory supplementation in a mouse model, and it is still necessary to further explore the precise molecular mechanisms at the gene level based on in vitro cell culture models. For instance, we can employ gene interference or knockout techniques to validate the involvement of NF-κB-mediated anti-inflammatory mechanisms in Cory’s protective effects against colistin-induced cytotoxicity and nephrotoxicity. Additionally, the pharmacokinetic characterization carried out during a pre-clinical study evaluates the drug’s absorption, distribution, metabolism, and elimination (ADME) [71]. It is very important to forecast its bioavailability and figure out the appropriate human dosage [71]. This is another limitation of this study. Moreover, based on the inherent properties of antibacterial drugs, further research is needed to determine whether Cory supplementation affects the antibacterial activity and therapeutic effect of colistin.

In summary, this investigation marks the initial discovery that Cory supplementation can effectively ameliorate kidney damage caused by colistin in a mouse model through its dual action on reducing oxidative damage and inflammatory responses. The underlying mechanisms may involve the suppression of cell death pathways related to mitochondria, modulation of the TGF-β/NOX4 axis, and regulation of the NF-κB signaling cascade, alongside the induction of the Nrf2/HO-1 antioxidant defense system.

## Data Availability

All data in the present study were included in this manuscript and the Supplementary Materials.

## Supplementary Materials

The following supporting information can be downloaded at: https://www.mdpi.com/article/10.3390/antiox14050593/s1, Supplementary Materials and Methods and Supplementary Table S1: Primer sequences of the quantitative real-time PCR.
