# Supplementary material for: Corynoxeine Supplementation Ameliorates Colistin-Induced Kidney Oxidative Stress and Inflammation in Mice
Source: Antioxidants (Basel). 2025 May 15;14(5):593. doi: 10.3390/antiox14050593 (PMC12108663; doi:10.3390/antiox14050593)
Supplement: Supplementary file 1 [file antioxidants-14-00593-s001.zip › antioxidants-3594094-supplementary.pdf]

Supplemental data

Corynoxeine supplementation ameliorates colistin-induced kidney oxidative stress and inflammation in mice

Yue Liu[1,2], Ruichen Zhang[1,2], Tony Velkov[3], Jianzhong Shen[1,2], Shusheng Tang[1,2*], Chongshan Dai[1,2*]

[1] National Key Laboratory of Veterinary Public Health and Safety, College of Veterinary Medicine, China Agricultural University, Beijing 100193, China.

[2] Technology Innovation Center for Food Safety Surveillance and Detection (Hainan), Sanya Institute of China Agricultural University, Sanya 572025, China.

[3] Department of Pharmacology, Biodiscovery Institute, Monash University, Parkville, VIC 3052, Australia.

*Correspondences: daichongshan@cau.edu.cn (C.D.); tssfj@cau.edu.cn (S.T.).

Suppl. Materials and Methods

2.4 Oxidative stress biomarker measurement

In this study, segments of renal tissue were carefully extracted and processed to create a 10% (w/v) homogenate, adhering to methodologies established in prior research [1]. The quantification of protein levels within the supernatant was determined using a bicin-choninic acid (BCA) Protein Assay Kit (Thermo Fisher Scientific, Massachusetts, USA). To assess oxidative stress parameters, measurements of the enzymatic activities of catalase (CAT) and superoxide dismutase (SOD) and malondialdehyde (MDA) and reduced glutathione (GSH) levels were conducted using specialized assay kits provided by the Nanjing Jiancheng Institute of Biological Engineering (Nanjing, China). All procedures were meticulously executed in compliance with the manufacturers' guidelines. A detailed protocol for each biomarker was shown below.

2.4.1 Measurement of MDA levels

All protocols were followed according to the instructions provided by the Reagent kit company (http://www.njjcbio.com/products.asp?id=287). In brief, kidney tissues (about 50 mg) were homogenized with 0.5 mL of Lysis buffer in a 1.5 mL size sterile Eppendorf tube using a High-Speed Low Temperature Tissue Grinding Machine (Sercivebio Company, Wuhan, China). After homogenate treatment, samples are centrifuged at 12,000 × g for 15 min at 4 °C, then add the reagents in sequence and allow them to react fully. The centrifuge tubes were heated at 95 °C for 40 min using a low-temperature linker. Upon completion of heating, the centrifuge tubes were cooled

in tap water and subsequently centrifuged at 4000 rpm for 10 minutes. Using a pipette, carefully transfer 200 μL of the supernatant, and measure the absorbance of each well at 530 nm with a microplate reader using a multi-wavelength microplate reader (Molecular Devices, Sunnyvale, CA, USA). Additionally, aliquot a portion of the supernatant to determine the protein concentration using a BCA™ protein assay kit.

2.4.2 Measurement of GSH levels

All protocols were followed according to the instructions provided by the Reagent kit company (http://www.njjcbio.com/products.asp?id=1532). In brief, approximately 20 mg of kidney tissue were added to physiological saline at a weight-to-volume ratio of 1:9 (g/mL) to prepare a tissue homogenate. After homogenization, centrifuge the sample at 2500 rpm for 10 minutes, and collect the supernatant. Transfer 0.1 mL of the supernatant into a new tube, add an equal volume of Reagent 1, mix thoroughly, and centrifuge at 3500 rpm for 10 minutes. Collect the resulting supernatant for subsequent colorimetric analysis. Follow the manufacturer's instructions to sequentially add the required reagents, ensuring complete reaction. Thoroughly mix the reagents, allow the mixture to stand for 5 minutes, then measure absorbance values at 450 nm using a multi-wavelength microplate reader (Molecular Devices, Sunnyvale, CA, USA). Additionally, the protein concentration of the supernatant was determined using a BCA™ protein assay kit.

2.4.3 Measurement of SOD activities

All protocols were followed according to the instructions provided by the Reagent kit company (http://www.njjcbio.com/products.asp?id=286). Briefly, approximately 20

mg of kidney tissue was homogenized in physiological saline at a weight-to-volume ratio of 1:9 (g/mL). The homogenate was centrifuged at 3000 rpm for 10 minutes, and the supernatant was collected. Following the manufacturer's protocol, the required reagents were sequentially added with thorough mixing to ensure complete reaction. After mixing, the 96-well plate was incubated at 37°C for 20 minutes. Absorbance values were then measured at 450 nm using a multi-wavelength microplate reader (Molecular Devices, Sunnyvale, CA, USA). Additionally, the protein concentration of the supernatant was determined using a BCA™ protein assay kit.

2.4.4 Measurement of CAT activities

All protocols were followed according to the instructions provided by the Reagent kit company (http://www.njjcbio.com/products.asp?id=286). Briefly, approximately 20 mg of kidney tissue was mixed with physiological saline at a weight-to-volume ratio of 1:9 (g/mL) to prepare a tissue homogenate. The mixture was homogenized and centrifuged at 4000 rpm for 10 minutes. The resulting supernatant was collected, and reagents were sequentially added following the manufacturer's protocol to ensure complete reaction. After thorough mixing, absorbance values were then measured at 450 nm using a multi-wavelength microplate reader (Molecular Devices, Sunnyvale, CA, USA). Additionally, the protein concentration of the supernatant was determined using a BCA™ protein assay kit.

2.5 Measurement of the activities of caspases-9 and -3

The enzymatic activities of caspase-9 and caspase-3 in mice kidney tissues were quantified through the utilization of commercially available assay kits (Beyotime,

Beijing, China). The experimental procedures were meticulously executed in strict compliance with the manufacturer's established guidelines. Kidney tissues (about 50 mg) were homogenized with 0.1 mL of Lysis buffer in a 1.5 mL size sterile Eppendorf tube using a High-Speed Low Temperature Tissue Grinding Machine (Sercivebio Company, Wuhan, China). After homogenate treatment, samples were centrifuged at 16,000× g for 15 min at 4 °C. Collect the supernatant for the colorimetric reaction, then add the reagents in sequence according to the instructions and allow them to react fully. Place the 96-well plate in an incubator at 37 °C for 60 minutes of incubation. After incubation is complete, use a multi-wavelength microplate reader to measure the absorbance values in each well at a wavelength of 405 nm (Molecular Devices, Sunnyvale, CA, USA). The quantification of protein levels was performed utilizing a BCA™ protein determination kit (Beyotime, Beijing, China). All experimental results pertaining to caspase-9 and caspase-3 enzymatic activities were subsequently normalized based on the quantified protein concentrations detected in corresponding samples.

2.6. Histopathological examination

Parts of kidney tissue samples were isolated and fixed in a 4% paraformaldehyde solution (approximately 10 times the volume of the tissue sample). Following a 48-hour fixation period, the tissues were then dehydrated in graded ethanol (70%, 85%, 90%, and 100%), paraffin-embedded, and sectioned at a 5 μm thickness. Subsequently, hematoxylin–eosin staining was performed, and the stained slides were examined using an optical microscope (Leica Microsystems, Wetzlar, Germany). Histopathological

changes were scored using a semi-quantitative score (SQS), according to the previously published method [2]. In brief, the degree of kidney damage was divided into three grades, i.e., grade 1, mild acute tubular damage with tubular dilation, and scored as 1; grade 2, severe acute tubular damage with necrosis of tubular epithelial cells, or numerous tubular casts, and scored as 4; or grade 3, acute cortical necrosis/infarction of tubules and glomeruli with or without papillary necrosis, and scored as 10. The percentages of the kidney damages were scored as follows: <1% was scored as 0, 1% ~ <5% was sored as 1, 5%~ <10% was scored as 2, 10% ~ <20% was scored as 3, 20%~ <30% was scored as 4, 30% ~ <40% was scored as 5, and ≥40% was scored as 6. The overall scores were calculated by the product of percentage score × grade score. Finally, an SQS for renal histological changes was assigned as follows: SQS 0 = no significant change (overall score < 1), SQS + 1 = mild damage (overall score 1 to <15), SQS + 2 = mild to moderate damage (overall score 15 to <30), SQS + 3 = moderate damage (overall score 30 to <45), SQS + 4 = moderate to severe damage (overall score 45 to <60), and SQS + 5 = severe damage (overall score 60). The final damage degrees were showed as the SQS (mean ± SD).

2.7. Immunohistochemical examination

After conducting the histopathological evaluation as described earlier, the paraffin-embedded kidney sections were subjected to streptavidin–biotin–peroxidase staining for immunohistochemical assays, according to our published protocols [1]. In brief, kidney tissue sections were subjected to deparaffinization and dehydration in graded alcohol, followed by microwave antigen retrieval for another 15 min in 10 mM sodium

citrate buffer (pH = 6.0; Beyotime, Beijing, China). Endogenous peroxidase activity was removed by 3% hydrogen peroxide for 15 min. Then, slices were included with 5 % goat serum for 30 min at room temperature to block the nonspecific antigens. Rabbit polyclonal antibodies targeting NOX4 and TGF-β (diluted at 1:200; ProteinTech Group, Inc., Chicago, IL, USA) were utilized. Kidney tissue sections were incubated for overnight at 4 °C. After washing with PBS for 3 times, kidney tissue sections were incubated with biotin-labeled goat anti-rabbit IgG (1:200; Santa Cruz, USA) for 1 h at 37 °C. After further incubation with horseradish peroxidase (HRP)-labeled streptavidin (for 30 min at room temperature), the kidney sections were washed for 3 times using PBS, then incubated with diaminobenzidine (DAB) (Beyotime, China) for staining. Images were collected using a Leica microscope (Wetzlar, Germany). The quantitative analysis is followed according to a previous study [3], as follows: 10 non-overlapping fields were randomly scanned per tissue section of each sample to determine the relative area percentage of immunohistochemical expression levels of NOX4 and TGF-β in different groups. Morphological measurements and analyzed data were obtained using a microscope (Leica Microsystems GmbH, Germany).

Suppl. Tables

Suppl. Table 1. Primer sequences of the quantitative real-time PCR.

Gene name	Direction	Primer sequence (5′to 3′)
NF-κB	forward	5′-CAC TGT CTG CCT CTC TCG TCT-3′

	reverse	5′-AAG GAT GTC TCC ACA CCA CTG-3′
TNF-α	forward	5′- AGCCGATGGGTTGTACCTTG-3′
	reverse	5′- ATAGCAAATCGGCTGACGGT-3′
IL-1β	forward	5′- CCGTGGACCTTCCAGGATGA-3′
	reverse	5′- GGGAACGTCACACACCAGCA-3′
IL-6	forward	5′- AGGATACCACTCCCAACAGACCT-3′
	reverse	5′- CAAGTGCATCATCGTTGTTCATAC-3′
β-actin	forward	GCCCTGAGGCTCTTTTCCA
	reverse	GTTGGCATAGAGGTCTTTACGGAT

References:

1. Dai, C., J. Li, S. Tang, J. Li and X. Xiao. "Colistin-induced nephrotoxicity in mice involves the mitochondrial, death receptor, and endoplasmic reticulum pathways." *Antimicrob Agents Chemother* 58 (2014): 4075-85. 10.1128/aac.00070-14.
2. Dai, C., S. Tang, Y. Wang, T. Velkov and X. Xiao. "Baicalein acts as a nephroprotectant that ameliorates colistin-induced nephrotoxicity by activating the antioxidant defence mechanism of the kidneys and down-regulating the inflammatory response." *J Antimicrob Chemother* 72 (2017): 2562-69. 10.1093/jac/dkx185.
3. Shafik, M. S., A. Bishr, D. M. El-Tanbouly and A. S. Attia. "Modulation of mir-205/ egln2 by rosuvastatin mitigates colistin-induced nephrotoxicity in rats: Involvement of atf4/ chop and nrf2 pathways." *Biomed Pharmacother* 157 (2023): 114042. 10.1016/j.biopha.2022.114042.
